# Phenolic Profiles and Antioxidant Potential of *Nymphaea* and *Nelumbo* Species: Botanical Authentication, Redox-Modulating Mechanisms, and Translational Perspectives

**DOI:** 10.3390/antiox15101336

**Published:** 2026-10-16

**Authors:** Maria Czernicka, Sebastian Such

**Affiliations:** Department of Bioenergetics, Food Analysis and Microbiology, Faculty of Technology and Life Sciences, University of Rzeszów, Zelwerowicza 8B St., 35-601 Rzeszów, Poland

**Keywords:** *Nymphaea*, *Nelumbo*, phenolic compounds, botanical authentication, redox signaling, benzylisoquinoline alkaloids, safety assessment

## Abstract

Natural products are important sources of redox-active phytochemicals, but meaningful interpretation of their biological effects requires accurate botanical and chemical characterization. The vernacular label “lotus” is applied to botanically distinct taxa belonging to the genera *Nymphaea* (Nymphaeaceae) and *Nelumbo* (Nelumbonaceae), despite their substantial taxonomic and phytochemical divergence. This review critically evaluates authenticated evidence on botanical identity, phenolic and alkaloid composition, antioxidant activity, redox-modulating mechanisms, translational relevance, and safety across selected species of both genera. Particular emphasis is placed on organ-specific phytochemical specialization, the contrasting distribution of flavonoids and benzylisoquinoline alkaloids, and the limitations of comparing antioxidant activity across heterogeneous extraction and assay systems. Available mechanistic studies implicate interconnected antioxidant-response, inflammatory, metabolic, autophagy, and glucose-handling pathways, but the evidence remains predominantly preclinical. *Nelumbo nucifera* is substantially better characterized than *Nymphaea* species, especially with respect to alkaloid chemistry, pharmacology, and toxicology, whereas evidence for *Nymphaea* remains more fragmented and species dependent. Botanical authentication, chemical standardization, pharmacokinetic characterization, and preparation-specific safety assessment are therefore essential for meaningful translation. Overall, the therapeutic potential of *Nymphaea* and *Nelumbo* is scientifically plausible, but current evidence is insufficient for broad clinical generalization or interchangeable use of materials marketed as “lotus”.

## 1. Introduction

The vernacular term “lotus” is widely used in the ethnobotanical literature and commercial herbal markets for botanically distinct aquatic plants. In particular, *Nymphaea nouchali* var. *caerulea* (Savigny) Burtt Davy (Nymphaeaceae) and *Nelumbo nucifera* Gaertn. (Nelumbonaceae) belong to different genera and families and differ markedly in taxonomy, phytochemical composition, pharmacological properties, and safety considerations [1,2,3,4]. Nevertheless, materials derived from both taxa may be marketed under the same informal designation “lotus”, creating ambiguity in the interpretation of commercial claims and scientific findings [2,3,5]. Consequently, biological effects reported for poorly authenticated “lotus” preparations cannot necessarily be assigned to either genus with confidence.

Research on phenolic compounds in *Nymphaea* and *Nelumbo* species has expanded substantially because of their antioxidant potential and broader pharmacological relevance [5,6]. Recent analytical advances, particularly ultra-high-performance liquid chromatography–mass spectrometry (UHPLC-MS)-based profiling and integrated transcriptomic and metabolomic approaches, have revealed considerable species- and tissue-dependent variation in phenolic compounds, flavonoid glycosides, alkaloids, and other specialized metabolites [7,8]. Such variation suggests that antioxidant activity attributed broadly to “lotus” cannot be interpreted independently of botanical identity, plant organ, extraction conditions, and chemical composition.

The taxonomic distinction between the two genera is unambiguous. *Nymphaea* belongs to Nymphaeaceae, whereas *Nelumbo* represents Nelumbonaceae, and the genera differ substantially in vegetative and reproductive morphology [9]. Despite these well-established differences, substitution and mislabeling continue to occur in commercial preparations, complicating both quality control and interpretation of the scientific literature [5]. Their phytochemical divergence is particularly evident in alkaloid profiles, including pronounced differences in the abundance and distribution of nuciferine and related compounds [8,10,11]. Consequently, pharmacological findings obtained from inadequately authenticated materials may reflect taxonomic substitution, differences in plant part, or compositional heterogeneity rather than genuine species-specific effects.

Botanical authentication therefore represents a prerequisite for reproducible phytochemical and pharmacological research [12]. Morphological identification and voucher specimen deposition remain fundamental, but processed botanical materials frequently require complementary molecular and chemical approaches, including DNA-based identification and chromatographic fingerprinting [13,14]. The inconsistent application of such procedures has contributed to a fragmented evidence base in which species-specific effects, extract-dependent responses, and potential safety concerns are not always clearly distinguishable.

Against this background, the present review critically integrates authenticated evidence on the phytochemistry, antioxidant activity, molecular redox-modulating mechanisms, translational potential, and safety of selected *Nymphaea* and *Nelumbo* species. Particular emphasis is placed on linking botanical identity and tissue-specific chemical composition with biological activity, while distinguishing mechanistic and preclinical evidence from findings that are sufficiently developed to support clinical or regulatory interpretation. By combining taxonomic authentication, comparative phytochemistry, redox biology, and safety assessment, the review aims to clarify which conclusions can currently be supported at species level and where major evidence gaps remain.

## 2. Botanical Authentication and Taxonomic Framework

*Nymphaea* and *Nelumbo* are not closely related botanical equivalents despite their persistent grouping under the vernacular term “lotus”. *Nymphaea* belongs to Nymphaeaceae within Nymphaeales, whereas *Nelumbo* belongs to Nelumbonaceae within Proteales, reflecting substantial evolutionary divergence between the two lineages [15,16,17]. For the purposes of authentication, however, the practical consequence of this phylogenetic separation is more important than its evolutionary chronology: the two genera differ consistently in morphology, biosynthetic capacity, and phytochemical composition [1,5].

**Figure 1:**
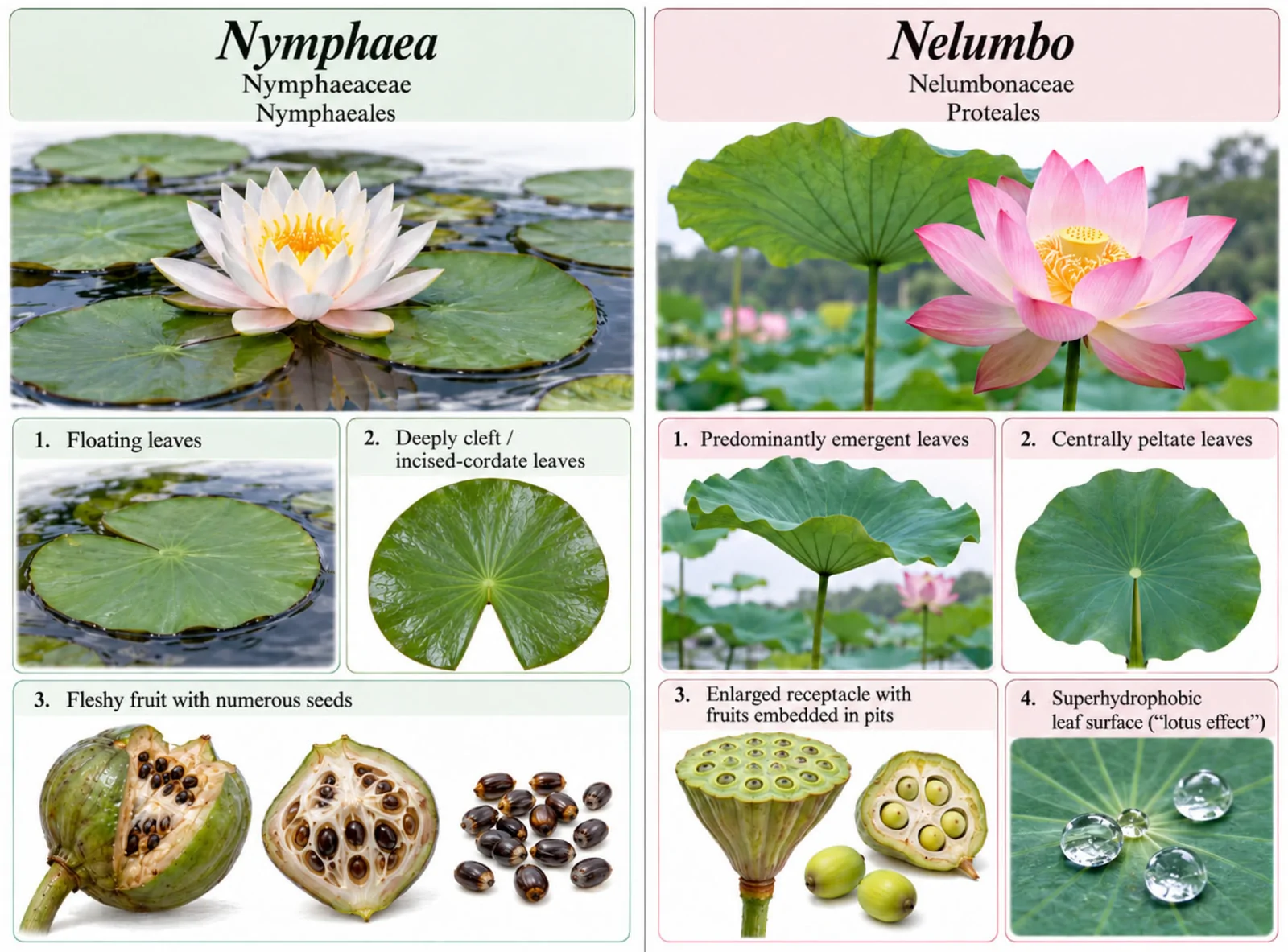
Principal taxonomic and morphological distinctions between *Nymphaea* and *Nelumbo*. The figure summarizes differences in family and order assignment, leaf habit and leaf morphology, reproductive structures, and the superhydrophobic leaf surface (“lotus effect”) characteristic of *Nelumbo*.

Morphological discrimination is generally straightforward in intact material. *Nymphaea* species typically produce floating, deeply cleft or incised-cordate leaves, whereas *Nelumbo* develops predominantly emergent, centrally peltate leaves [18,19]. Reproductive morphology provides an equally distinctive criterion: *Nelumbo* forms a characteristic enlarged receptacle with individual fruits embedded in pits, whereas *Nymphaea* develops fleshy fruits containing numerous, often arillate seeds [16,17]. The superhydrophobic leaf surface associated with the classical “lotus effect” is additionally characteristic of *Nelumbo* [20]. These distinctions make the continued commercial conflation of the genera difficult to justify botanically. More importantly, once intact diagnostic structures are removed during drying, milling, extraction, or formulation, morphological identification becomes progressively less reliable. Thus, morphology should be regarded as the first authentication layer rather than a sufficient stand-alone criterion for processed “lotus” products [21]. The principal botanical distinctions between *Nymphaea* and *Nelumbo*, including taxonomic affiliation and key morphological traits, are summarized in Figure 1.

DNA-based methods provide a complementary means of authentication when diagnostic morphology is unavailable [21,22]. In *Nymphaea*, the internal transcribed spacer (ITS) has shown strong species-level discriminatory power, while complementary plastid markers can improve resolution for taxa that cannot be reliably distinguished using a single locus [23]. Combined-marker approaches are therefore preferable when closely related taxa or processed botanical materials are examined. For complex herbal preparations, DNA metabarcoding can additionally reveal labeled and unlabeled botanical components that conventional single-species assays may overlook [14,24]. The reliability of these approaches nevertheless depends on the quality and taxonomic accuracy of reference sequence databases and appropriately authenticated reference material [21].

Chemical fingerprinting provides a second, functionally important authentication layer because it addresses not only botanical identity but also the composition of the material ultimately used in biological studies. High-performance liquid chromatography–quadrupole time-of-flight mass spectrometry (HPLC-QTOF-MS) and ultra-high-performance liquid chromatography coupled with photodiode array detection and electrospray ionization quadrupole time-of-flight mass spectrometry (UHPLC/PDA/ESI-QTOF-MS) approaches can resolve characteristic alkaloid and phenolic profiles and reveal substantial organ- and geographic-origin-dependent variability [7,25]. Nuciferine may be particularly informative as one component of a broader chemical fingerprint: authentic *N. caerulea* concretes and absolutes contained only trace amounts of nuciferine (10–72 ppb), whereas *N. nucifera* tissues have been reported to contain nuciferine at mg/g levels [2,26]. However, nuciferine should not be treated as a stand-alone taxonomic marker, because its abundance is strongly influenced by plant organ, extraction conditions, and sample composition.

Accordingly, the strongest authentication strategy is not morphological, molecular, or chemical identification alone, but their convergent use as complementary evidence. Voucher-supported taxonomy establishes the biological source, DNA analysis strengthens species assignment, and chromatographic or metabolomic fingerprinting verifies that the analyzed preparation retains a composition compatible with that assignment. This distinction is particularly important in pharmacological studies, where correct species identification does not by itself guarantee chemical comparability between extracts.

The consequences of inadequate authentication extend beyond nomenclatural imprecision. Studies reporting biological effects of generically designated “lotus” preparations without voucher documentation or sufficiently detailed analytical characterization cannot be confidently assigned at species level [27,28]. This creates a circular problem in the literature: poorly authenticated material generates apparently species-specific pharmacological claims, which may subsequently be cited as evidence for the same species despite uncertainty concerning the original botanical source.

The problem becomes more serious when inadequate authentication overlaps with commercial adulteration or undeclared psychoactive constituents. Commercial “blue lotus” products have been shown to differ substantially from authenticated *N. caerulea* extracts in chemical composition [2], while forensic investigations identified synthetic cannabinoids in products marketed or described as “blue lotus” [29]. These findings demonstrate that adverse or psychoactive effects associated with products marketed as “blue lotus” cannot automatically be interpreted as intrinsic properties of authenticated *N. caerulea*. Historical discussions of the pharmacological properties of the Egyptian lotus should therefore be interpreted as historical context rather than direct evidence for the composition or safety of contemporary authenticated preparations [30].

From a critical-review perspective, botanical authentication should be treated as a criterion of evidence quality rather than merely as a methodological prerequisite. Studies based on voucher-authenticated specimens and chemically characterized extracts provide a stronger basis for species-level inference than reports relying on vernacular product names alone. Without verified identity and adequate chemical characterization, apparent differences in antioxidant activity, pharmacological efficacy, or safety may instead reflect substitution, organ-specific chemistry, extraction-dependent variation, or adulteration. Studies lacking adequate authentication should therefore carry less weight in comparative interpretation, even when their biological outcomes appear compelling.

## 3. Phenylpropanoid Biosynthetic Pathways

**Figure 2:**
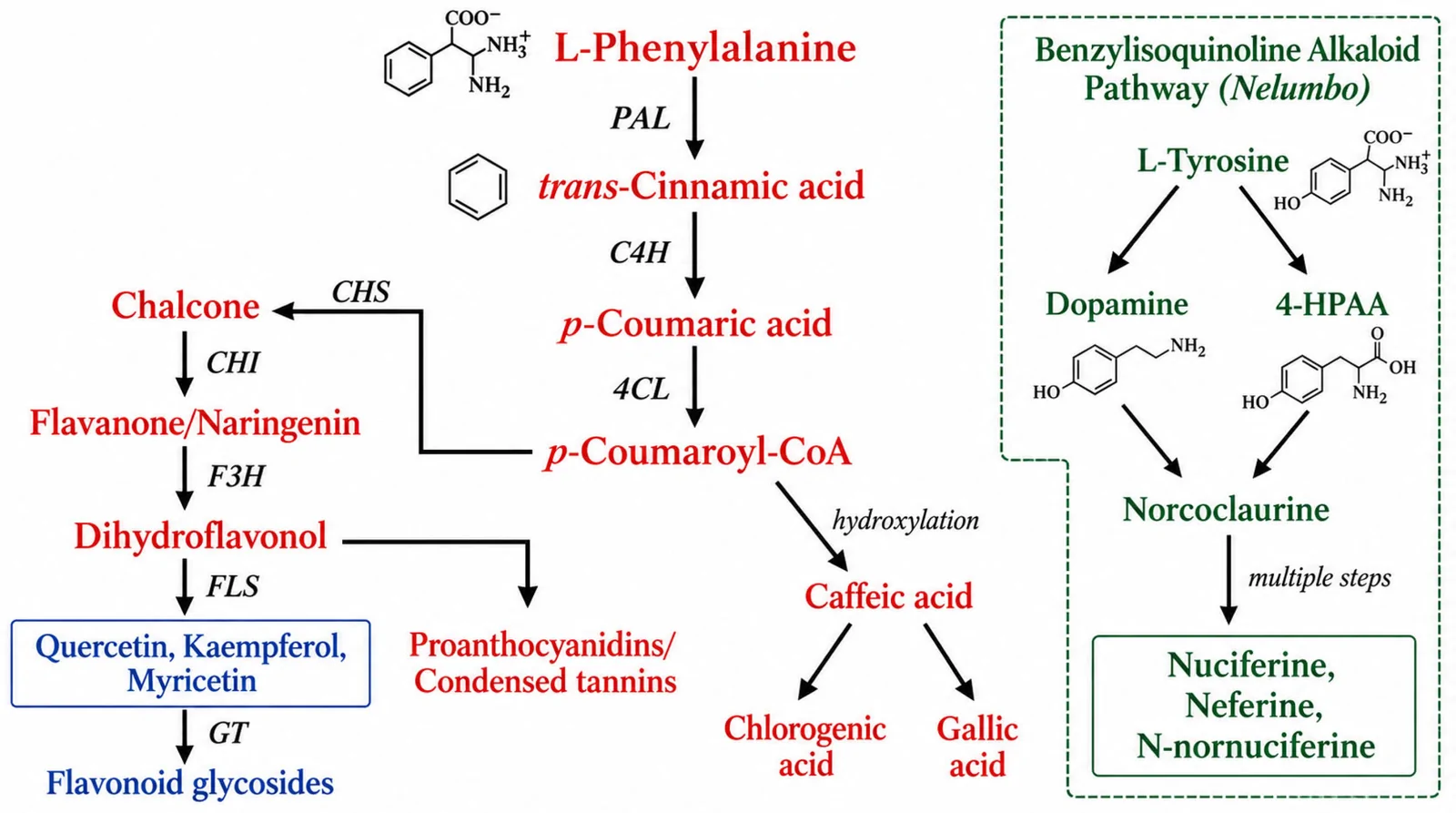
Simplified biosynthetic framework illustrating the shared phenylpropanoid pathway and major downstream phytochemical branches relevant to *Nymphaea* and *Nelumbo*. The scheme highlights the common phenylpropanoid route leading to flavonoids and phenolic acids and the specialized benzylisoquinoline alkaloid pathway associated with *Nelumbo*, including representative nuciferine- and neferine-related products. The diagram is intended as a comparative pathway overview rather than a complete reconstruction of all enzymatic steps or species-specific intermediates. Enzyme abbreviations: PAL, phenylalanine ammonia-lyase; C4H, cinnamate 4-hydroxylase; 4CL, 4-coumarate:CoA ligase; CHS, chalcone synthase; CHI, chalcone isomerase; F3H, flavanone 3-hydroxylase; FLS, flavonol synthase; GT, glycosyltransferase.

The phenylpropanoid pathway provides a common biosynthetic framework for the phenolic diversity of *Nymphaea* and *Nelumbo*, although downstream allocation of phenylpropanoid-derived metabolites differs substantially among genera, species, and tissues. Phenylalanine ammonia-lyase (PAL), cinnamate 4-hydroxylase (C4H), and 4-coumarate:CoA ligase (4CL) constitute the general phenylpropanoid pathway, converting phenylalanine to p-coumaroyl-CoA, a central precursor for flavonoids, hydroxycinnamate derivatives, and related phenolics [31]. Comparative metabolomic studies further demonstrate pronounced organ- and tissue-dependent specialization in both genera, including distinct metabolite profiles among *Nymphaea nouchali* organs and differential flavonoid accumulation among *N. nucifera* tissues [7,32]. In *N. nucifera*, transcriptomic analyses additionally show developmental regulation of flavonoid and anthocyanin biosynthetic genes, particularly during petal development and color transition [33]. Thus, the shared upstream phenylpropanoid pathway itself is less informative for distinguishing the two genera than the differential regulation and diversification of downstream phenolic branches. The major points of convergence and divergence are summarized in Figure 2, which places the shared phenylpropanoid core and its flavonoid branch alongside the distinct alkaloid biosynthetic route relevant to the contrasting phytochemical profiles of *Nymphaea* and *Nelumbo*.

Downstream flavonoid metabolism gives rise to quercetin-, kaempferol-, myricetin-, luteolin-, and related glycosylated derivatives, but their relative abundance and conjugation patterns differ markedly among species and tissues [5,7]. Importantly, the biologically relevant distinction is not simply the presence of the broadly conserved flavonoid pathway, but the extent and pattern of its downstream specialization. In *Nelumbo nucifera*, C-glycosylated flavones are particularly enriched in the seed embryo (plumule), where both mono- and di-C-glycosides accumulate; dedicated C-glycosyltransferases involved in their stepwise formation have also been functionally characterized [34,35,36]. By contrast, *Nymphaea* tissues show diverse profiles dominated by flavonol glycosides and other phenolic conjugates, with pronounced organ- and population-dependent variation [7,37]. C-glycosylation also confers greater resistance to glycosidase- and acid-mediated hydrolysis than O-glycosylation, which may enhance the chemical persistence of these derivatives in plant tissues and processed preparations [38]. This distinction is important when antioxidant potential is inferred from bulk phytochemical indices. Total phenolic or flavonoid content alone cannot resolve differences in substitution pattern, glycosylation, redox reactivity, or metabolic stability and therefore provides only a partial representation of the functional phytochemical profile. Consequently, apparently similar total phenolic content (TPC) or total flavonoid content (TFC) values across species or tissues should not be interpreted as evidence of equivalent antioxidant capacity without complementary compositional characterization.

A more consequential biosynthetic divergence concerns alkaloid metabolism. *N. nucifera* possesses a specialized benzylisoquinoline alkaloid network in which L-tyrosine-derived dopamine and 4-hydroxyphenylacetaldehyde condense to form (R,S)-norcoclaurine. Downstream O- and N-methyltransferases and cytochrome P450 enzymes preferentially channel R-configured intermediates toward proaporphine, aporphine, and bisbenzylisoquinoline scaffolds, yielding major alkaloids such as nuciferine, neferine, liensinine, and isoliensinine [39,40,41]. Cytochrome P450 family 80 (CYP80) enzymes participate in key oxidative coupling reactions underlying this structural diversification, including the formation of proaporphine and bisbenzylisoquinoline scaffolds [39,42]. This specialized pathway provides a strong mechanistic basis for the distinctive alkaloid profile of *N. nucifera*.

However, the distinction should not be simplified to the presence of alkaloids in *Nelumbo* and their absence from *Nymphaea*. Alkaloids have also been reported across the genus *Nymphaea*, although their composition is heterogeneous and may include classes distinct from the benzylisoquinoline-dominated profile characteristic of *N. nucifera* [1,7]. The comparative phytochemical distinction therefore lies primarily in compound identity and pathway architecture rather than in total alkaloid abundance per se. This is particularly important when crude measurements of “total alkaloids” are compared with quantitative determinations of individual aporphine and bisbenzylisoquinoline alkaloids, such as nuciferine, neferine, liensinine, and isoliensinine, because these analytical categories are not chemically equivalent [43].

Biosynthetic divergence is further amplified by tissue-specific pathway regulation. In *N. nucifera*, leaves preferentially accumulate flavonoid O-glycosides and aporphine alkaloids, whereas seed coats and seedpods are particularly rich in proanthocyanidins/procyanidins. The seed embryo (plumule), by contrast, is enriched in C-glycosylated flavonoids and represents a major site of bisbenzylisoquinoline alkaloid accumulation, including neferine, liensinine, and isoliensinine [34,40,44,45]. *Nymphaea* species likewise exhibit pronounced organ-specific metabolite profiles: comparative UHPLC/PDA/ESI-QTOF-MS profiling of *N. nouchali* demonstrated substantial differences among flowers, leaves, and stems in flavonoids, phenolic acids, ellagitannins, anthocyanins, and alkaloids (Younis et al., 2025) [7]. Recent integrated transcriptomic and metabolomic analyses of *N. nucifera* further support tissue-specific pathway regulation and gene-expression patterns as major determinants of metabolite specialization [32].

Accordingly, the phytochemical distinction between *Nymphaea* and *Nelumbo* should not be reduced to differences in the concentrations of individual compounds. It reflects differences in downstream enzymatic specialization, pathway regulation, and tissue-specific metabolic allocation. This biosynthetic perspective is essential for interpreting the quantitative profiles discussed below, because apparent differences in antioxidant or pharmacological activity may arise not simply from “more” or “less” phenolic material, but from fundamentally different mixtures of phenolics and alkaloids with distinct chemical and biological properties.

## 4. Phytochemical Profiles by Species

### 4.1. Phytochemical Diversity Within “Nymphaea”

*Nymphaea* species share a phenolic-rich phytochemical background but differ substantially in the relative contribution and structural diversity of flavonoids, phenolic acids, alkaloids, anthocyanins, and other specialized metabolites [1,37,46]. In *N. nouchali* var. *caerulea* (syn. *N. caerulea*), phytochemical studies have consistently demonstrated a prominent contribution of flavonoids and phenolic acids. Flower-derived material contains numerous quercetin-, kaempferol-, and myricetin-derived glycosides together with gallic and *p*-coumaric acids [11]. Younis et al. [7] substantially expanded this profile using UHPLC/PDA/ESI-QTOF-MS, annotating 185 metabolites across authenticated Egyptian flower, leaf, and stem material, including 58 flavonoids, 23 phenolic acids, 26 ellagitannins, seven anthocyanins, and six alkaloids. Earlier, Alam et al. [9] identified 54 secondary metabolites in *N. nouchali* stem by high-resolution mass spectrometry (HRMS), with phenolic acids and flavonoids predominating. Collectively, these findings indicate that the chemical identity of blue-lotus material cannot be adequately represented by a small set of conventional flavonoid markers and instead requires organ-specific and analytically resolved phytochemical profiling.

Particularly important is the interpretation of nuciferine. Dosoky et al. [2] detected only trace concentrations of nuciferine (10–72 ppb) in authenticated *N. caerulea* concretes and absolutes, while apomorphine was not detected. These values should be interpreted in the context of the specific extraction procedure used, but they contrast sharply with the quantitatively prominent occurrence of nuciferine in *N. nucifera*, where mg/g-level concentrations have been reported in selected tissues [25,26]. This difference is analytically useful, but trace detection should not be confused with pharmacological relevance. Claims concerning nuciferine-mediated effects of *N. caerulea* preparations therefore require particular caution unless the compound has been quantitatively demonstrated in the material actually tested.

Other *Nymphaea* species further illustrate why genus-level generalization is problematic. *N. lotus* floral tissues are rich in flavonol glycosides, particularly derivatives of kaempferol, quercetin, myricetin, and isorhamnetin [37], while crude phytochemical analysis of the rhizome has also reported high total alkaloid content without compound-level identification [47]. These data are particularly relevant because total alkaloid abundance should not be equated with the benzylisoquinoline profile characteristic of *Nelumbo*. In *N. rubra*, optimized ultrasonic extraction yielded high total phenolic and flavonoid contents and enabled high-resolution MS annotation of a broad secondary-metabolite profile [46]. *N. alba*, in turn, has been characterized by a phytochemical profile rich in hydrolysable tannins, gallic-acid derivatives, and flavonoid glycosides [48]. Collectively, these examples reinforce that phytochemical interpretation within *Nymphaea* should remain species-, organ-, and method-specific.

Direct ranking of these species according to TPC or TFC is nevertheless problematic. Published values originate from different plant organs, extraction systems, analytical procedures, calibration standards, and reporting units, while geographic variation adds another source of heterogeneity [37]. The apparent phytochemical hierarchy within *Nymphaea* is therefore partly biological and partly methodological, and numerical differences between studies should not automatically be interpreted as intrinsic species-level superiority.

### 4.2. Organ-Specific Phytochemical Specialization in Nelumbo nucifera

Compared with the relatively heterogeneous evidence available across *Nymphaea* species, *N. nucifera* is characterized by a more extensively documented pattern of organ-specific phytochemical specialization. Leaves contain abundant flavonoid glycosides, with quercetin derivatives among the predominant constituents, together with a chemically diverse aporphine alkaloid fraction that includes nuciferine and N-nornuciferine [25,49]. Flowers and stamens likewise contain diverse flavonoid and flavonol glycosides, and recent analytical and fractionation studies have further expanded the characterized phenolic profile of these tissues [37,50]. Importantly, the phenolic abundance reported for lotus organs should not be interpreted independently of developmental stage, geographic origin, extraction procedure, and analytical platform.

Seed-associated tissues constitute chemically distinct matrices, but should not be treated as a single phytochemical compartment. Seed embryos are particularly enriched in C-glycosylated flavonoids, whereas seed coats and seedpods contain substantial proanthocyanidin/procyanidin fractions and other phenolics [34,45]. Zhao et al. [51] further demonstrated that the concentrations of phenolic and alkaloid constituents in lotus seed and rhizome extracts vary with growing region and sample condition, underscoring that species-level authentication does not guarantee phytochemical equivalence among preparations.

**Figure 3:**
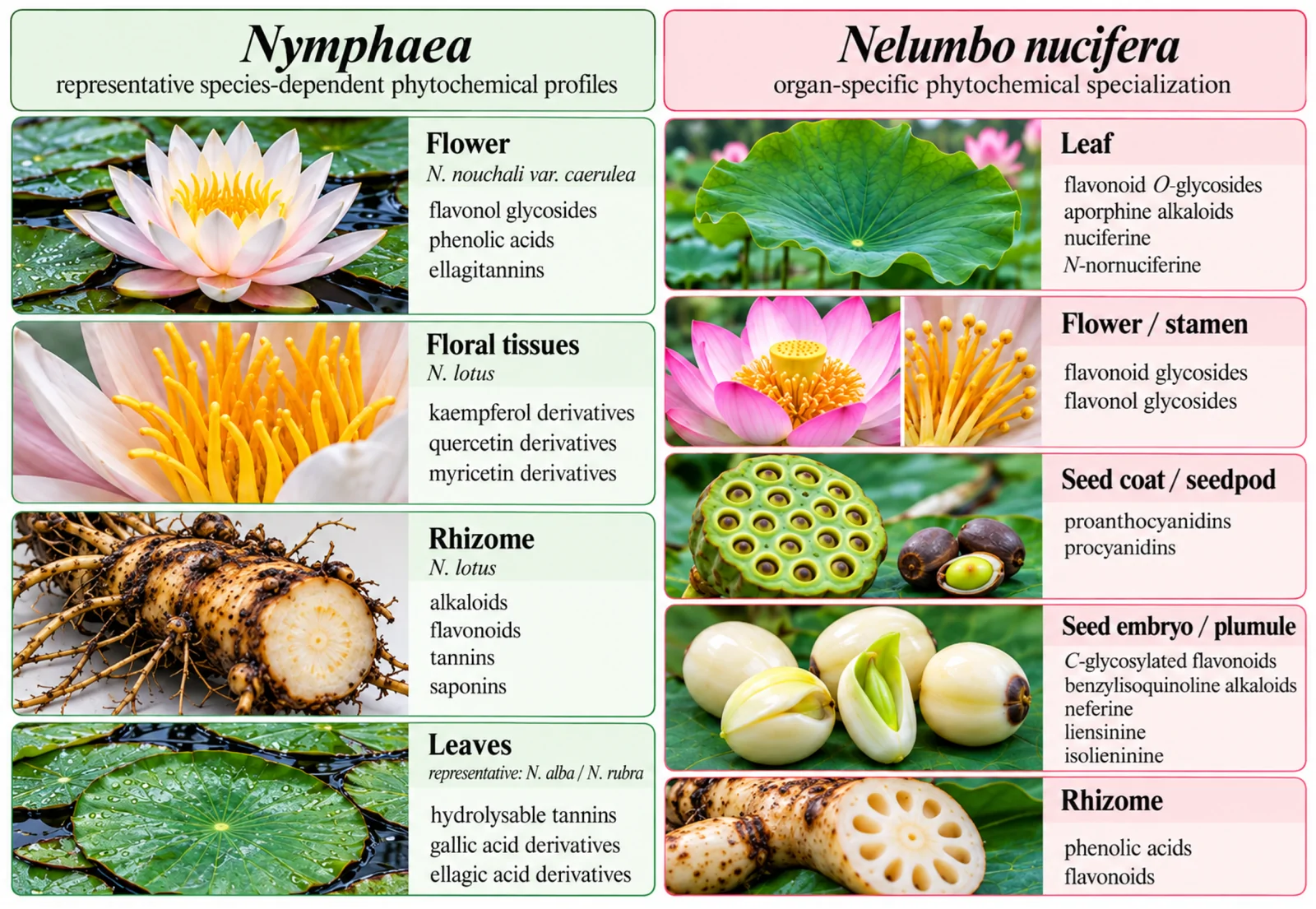
Representative species- and organ-dependent phytochemical profiles of *Nymphaea* taxa and *Nelumbo nucifera*. The scheme qualitatively summarizes the predominant phytochemical classes reported in selected organs and tissues, highlighting the comparatively heterogeneous, species-dependent profiles within *Nymphaea* and the more clearly documented organ-specific specialization of *N. nucifera*. Botanical identity and tissue origin therefore define chemically distinct phytochemical interventions, and materials described broadly as “lotus extract” should not be regarded as chemically uniform. The figure is intended as a qualitative synthesis and does not imply quantitative ranking among taxa, organs, or compound classes.

The seed embryo (plumule) represents an especially distinctive compartment because it is a major source of the bisbenzylisoquinoline alkaloids neferine, liensinine, and isoliensinine [40,52]. This contrasts with lotus leaves, in which aporphine alkaloids such as nuciferine and nornuciferine are prominent [25,49]. Tissue identity may therefore be nearly as important as species identity when interpreting pharmacological evidence. Accordingly, “*N. nucifera* extract” is not a chemically sufficient experimental descriptor unless the plant organ and extraction procedure are specified. Leaf, seed coat, embryo, flower, stamen, and rhizome preparations may represent fundamentally different phytochemical interventions rather than interchangeable preparations of the same botanical species. These contrasting species- and organ-dependent patterns of phytochemical specialization in *Nymphaea* and *N. nucifera* are summarized in Figure 3.

### 4.3. Comparative Phytochemical Patterns

The quantitative ranges reported across species and organs are summarized in Table 1. Because the underlying studies differ in extraction procedure, analytical platform, calibration standard, geographic origin, and reporting units, these values should be interpreted primarily as comparative ranges rather than as a formal quantitative ranking of species.

Interpretive note. Total phenolic content (TPC) or total flavonoid content (TFC) values in this table are method-dependent descriptors rather than species-level rankings. Alkaloid values reported as “total alkaloids” should not be equated with compound-specific benzylisoquinoline or aporphine concentrations. Several comparative patterns nevertheless emerge from the available evidence. First, *N. nucifera* shows the most clearly documented organ-specific partitioning of phenolics and alkaloids, whereas the available *Nymphaea* dataset is less systematic and substantially more heterogeneous across species and experimental designs [1,7,41]. Second, alkaloid identity may provide greater discriminatory value than bulk TPC or TFC because structurally distinct alkaloid classes characterize different taxa and tissues [25,40,57]. Third, geographic origin, season, organ identity, extraction conditions, and analytical methodology introduce substantial within-species variation that may confound direct comparisons between taxa [37,46,51,53].

**Figure 4:**
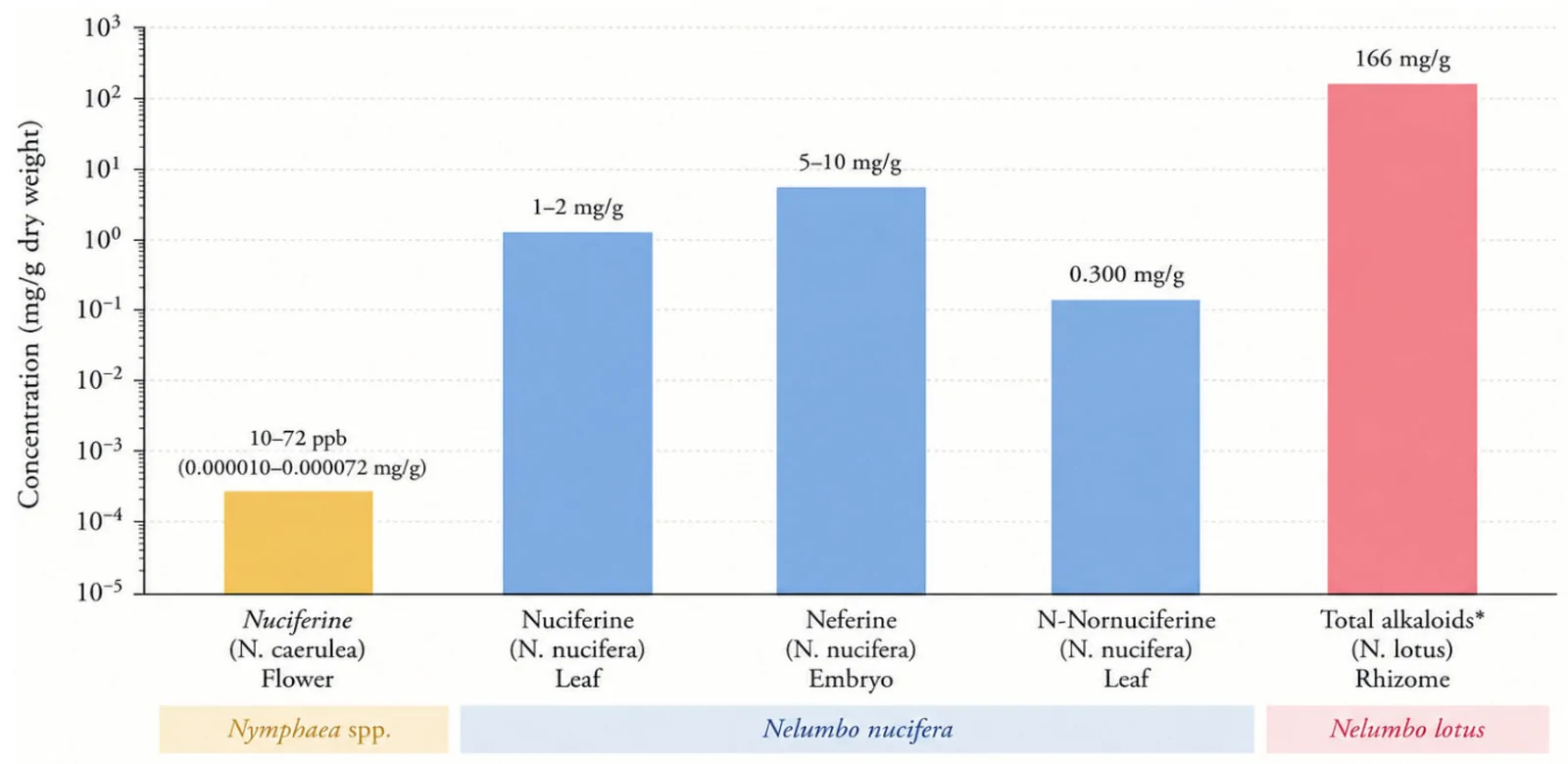
Reported alkaloid concentrations in selected *Nymphaea* and *Nelumbo* materials on a logarithmic scale. Bars show reported concentration ranges, except total alkaloids in *N. lotus* rhizome, presented as mean ± SD. The figure highlights the marked differences in alkaloid abundance between trace nuciferine in authenticated *N. caerulea* flower concretes/absolutes and bisbenzylisoquinoline alkaloids in *N. nucifera* embryo/plumule. Total alkaloid content is not directly comparable with concentrations of individual alkaloids. Data were compiled from studies cited in the main text and Table 1. The asterisk (*) indicates the total alkaloid content determined using a class-level assay, rather than the concentration of an individual alkaloid. These values are not directly comparable.

Accordingly, phytochemical differentiation should be based on multidimensional chemical fingerprints rather than isolated concentration values. High TPC or TFC does not establish phytochemical or antioxidant superiority, and the presence of a pharmacologically interesting compound does not establish its biological relevance unless its concentration, chemical context, and achievable exposure are considered. Indeed, antioxidant capacity may reflect complex combinations of phytochemicals rather than the contribution of a single constituent [37]. This distinction is particularly evident for alkaloids, for which reported concentrations span several orders of magnitude and reveal differences that are obscured when compounds are discussed only qualitatively (Figure 4).

The alkaloid comparison reinforces the distinction between chemical detection and biological significance. Trace nuciferine reported in authenticated *N. caerulea* flower concretes and absolutes, and the markedly higher concentrations of distinct bisbenzylisoquinoline alkaloids in *N. nucifera* seed embryos/plumules, represent qualitatively different exposure scenarios [2,35,40]. Compound identity, concentration, plant organ, extraction procedure, and matrix composition should therefore be considered jointly when relating phytochemical composition to biological activity. These compositional differences provide the necessary context for interpreting antioxidant activity. Importantly, the transition from phytochemical abundance to functional antioxidant effects is not linear: structurally different flavonoids, phenolic acids, and alkaloids may differ in redox reactivity, stability, bioaccessibility, and cellular activity even when bulk TPC or TFC values appear comparable. The following section therefore evaluates antioxidant evidence at the level of assay response and mechanism rather than assuming biological activity directly from phytochemical abundance.

## 5. Antioxidant Activity: Chemical Basis, Complementary Assays, and Cellular Protection

### 5.1. Chemical Basis of Antioxidant Activity

The antioxidant activity reported for *Nymphaea* and *Nelumbo* preparations reflects the combined contribution of chemically diverse phenolics, flavonoids, alkaloids, and other redox-active constituents rather than a single dominant compound class. Structural features such as the number and position of hydroxyl groups, B-ring substitution, conjugation within the flavonoid skeleton, and glycosylation pattern influence electron donation, hydrogen atom transfer, radical stabilization, and metal-binding capacity [58,59]. Flavonols with greater B-ring hydroxylation, particularly quercetin- and myricetin-type structures, generally show stronger intrinsic radical-scavenging potential than less hydroxylated analogues such as kaempferol, although these relationships are substantially modified by conjugation, matrix composition, and extract processing [58].

Glycosylation is particularly relevant because major flavonoids identified in both genera frequently occur as conjugated rather than free aglycone forms [7,37]. O-glycosylation can reduce direct radical-scavenging activity relative to the corresponding aglycone, although its effects on stability, absorption, and biological activity are context-dependent [60]. By contrast, C-glycosylation confers greater resistance to enzymatic and acid hydrolysis and can therefore enhance chemical persistence [38]. Consequently, antioxidant potential cannot be inferred reliably from TPC or TFC alone, because extracts with similar bulk phenolic values may contain chemically distinct compounds with different redox reactivity, stability, and bioaccessibility.

This limitation is particularly important for cross-species comparisons. The phytochemical heterogeneity documented in Section 4 means that measured antioxidant activity reflects not only botanical origin but also plant organ, extraction selectivity, compound conjugation, and assay chemistry. Accordingly, antioxidant assays should be interpreted as complementary analytical responses rather than as direct measures of in vivo antioxidant efficacy.

### 5.2. Antioxidant Activity Across Complementary Assay Systems

Available studies generally support substantial radical-scavenging and reducing activity in extracts from both genera, although direct comparison is constrained by differences in extraction procedure, assay conditions, concentration expression, and reference standards. *N. rubra* leaf extracts showed measurable activity across DPPH, ABTS, and FRAP assays, with optimized ultrasonic extraction substantially enhancing antioxidant responses [46]. In *N. nucifera*, antioxidant activity likewise varies markedly with plant organ and extraction or fractionation procedure. Stamen and perianth extracts from geographically distinct populations exhibited substantial DPPH, ABTS, and FRAP activities [37], whereas sequentially fractionated flower extracts showed pronounced fraction-dependent differences across all three assay systems, with the 30% ethanol fraction displaying the strongest activity [50]. Earlier work on *N. nucifera* leaves also demonstrated greater DPPH-scavenging activity in ethyl acetate and *n*-butanol fractions than in the aqueous fraction [61]. These findings are consistent with the substantial phenolic abundance reported for several *N. nucifera* tissues, but they do not establish intrinsic species-level superiority because plant organ, extraction procedure, season, and assay conditions differ considerably among studies [53].

FRAP measurements likewise demonstrate substantial reducing capacity, although the magnitude of the response varies markedly with plant organ and extraction procedure. In *N. lotus* stamens, ultrasonic-assisted extraction followed by macroporous-resin enrichment substantially increased DPPH, ABTS, and FRAP responses relative to less selective extraction procedures [62]. In *N. nucifera*, Zhu et al. [56] showed pronounced tissue-specific differences among seed and rhizome fractions: the seed embryo exhibited the highest DPPH and ABTS activities, whereas the rhizome knot showed the highest FRAP response. Lipid-peroxidation models provide another complementary endpoint. A phenolic-rich fraction from *N. nucifera* leaves inhibited thiobarbituric acid reactive substances (TBARS) formation in a linoleic-acid emulsion and reduced membrane lipid peroxidation in cultured hepatocytes [63], whereas hydroalcoholic *N. nouchali* seed extract inhibited TBARS formation in a concentration-dependent manner [64]. Recent organ-level comparisons within *Nymphaea* further confirm that apparent antioxidant activity can differ substantially among stamens, petals, and leaves even within closely related cultivars [65].

However, the different assays probe overlapping but non-equivalent chemical properties. DPPH and ABTS largely reflect radical-scavenging capacity under defined chemical conditions, while FRAP measures reducing power, whereas TBARS-based approaches assess inhibition of lipid oxidation. Their numerical outputs should therefore not be combined into a single hierarchy of “antioxidant strength”. In particular, differences in solvent polarity, assay pH, radical accessibility, calibration standard, extraction selectivity, and reporting units can substantially influence the apparent ranking of extracts. The available data are summarized in Table 2, which should therefore be interpreted as a comparative overview of reported assay responses rather than as a standardized ranking of species or extracts.

The assay literature supports antioxidant potential across both genera but remains analytically heterogeneous. Accordingly, the strongest conclusion is not that one species exhibits intrinsically greater antioxidant activity than another, but that phytochemical composition, extraction selectivity, and assay methodology strongly shape the measured response.

### 5.3. Cellular Antioxidant Protection and Biological Relevance

Chemical antioxidant assays provide useful screening information but do not reproduce cellular uptake, metabolism, redox signaling, or intracellular compartmentalization. Cellular models therefore offer an important intermediate level of evidence between cell-free antioxidant capacity and the molecular signaling mechanisms discussed in Section 6.

Ultrasound-assisted cellulase extraction of *Nymphaea hybrid* flowers was optimized to recover flavonoids and polyphenols, and subsequent macroporous-resin purification yielded an enriched extract capable of reducing reactive oxygen species (ROS) accumulation in H_2_O_2_-challenged HaCaT keratinocytes [33]. Complementary evidence from *N. nucifera* leaves showed that a phenolic-rich fraction increased cell viability and suppressed intracellular ROS and membrane lipid peroxidation in H_2_O_2_-challenged hepatocytes [66]. These findings illustrate how extract composition and processing can influence not only measured phytochemical yield but also subsequent cellular activity. Nevertheless, extraction procedure should be regarded as a determinant of extract composition and bioactivity rather than as an antioxidant mechanism in its own right.

Reduced intracellular ROS may arise from direct chemical quenching, modulation of endogenous antioxidant enzymes, suppression of pro-oxidant pathways, or activation of stress-response signaling. Cellular antioxidant protection therefore cannot identify mechanism by itself and requires pathway-level evidence, providing the transition to the molecular redox mechanisms discussed below.

## 6. Molecular Redox-Modulating Mechanisms

**Figure 5:**
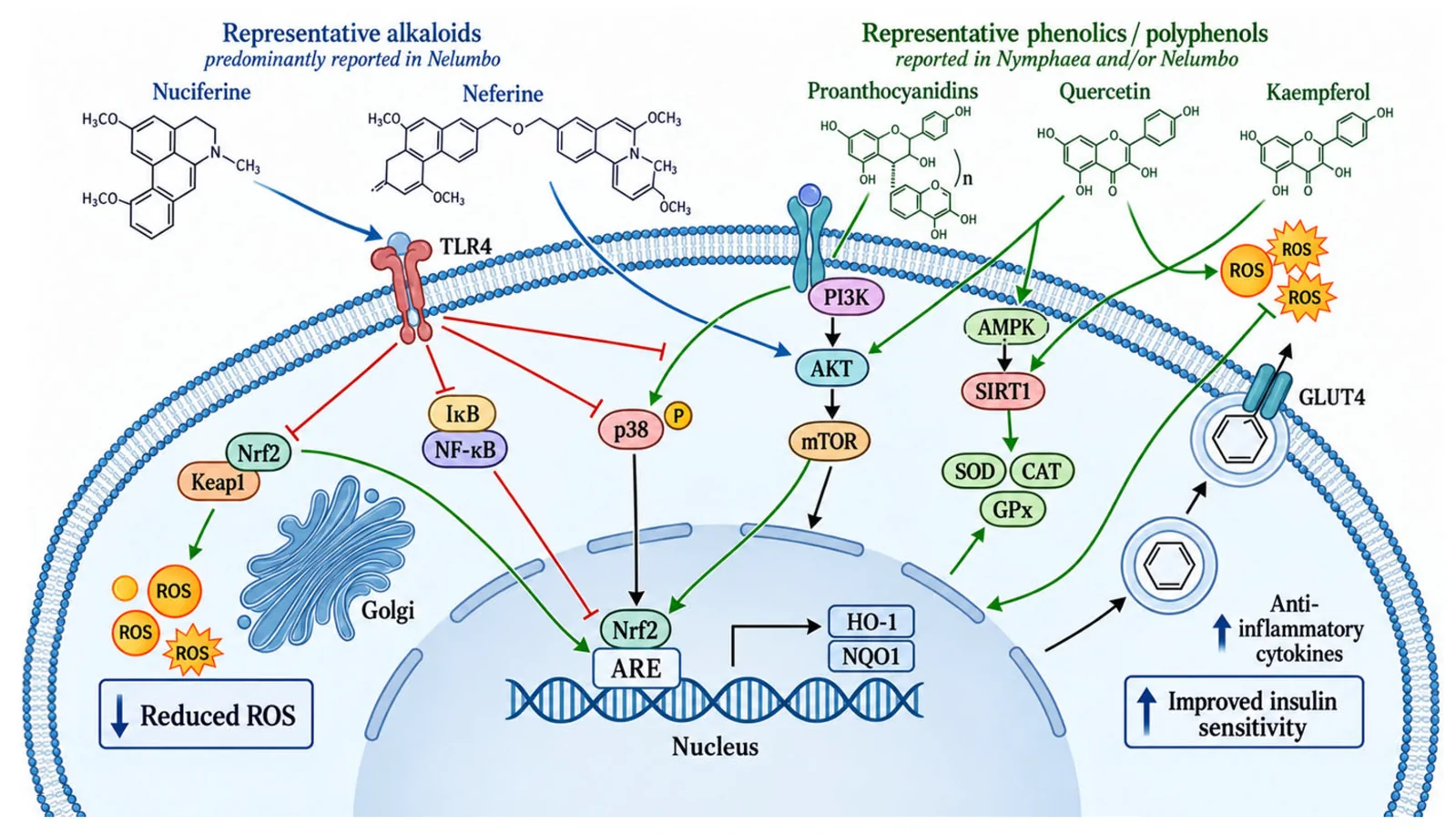
Integrated redox-modulating pathways associated with representative bioactive constituents reported in *Nymphaea* and *Nelumbo* species. The scheme summarizes major preclinical mechanisms involving Nrf2/Keap1/ARE, TLR4/NF-κB/MAPK, PI3K/AKT/mTOR, AMPK/SIRT1, antioxidant enzymes, and GLUT4-associated glucose handling. Arrows indicate reported or proposed pathway relationships and do not necessarily imply direct molecular binding. ROS, reactive oxygen species; ARE, antioxidant response element; HO-1, heme oxygenase-1; NQO1, NAD(P)H quinone dehydrogenase 1; SOD, superoxide dismutase; CAT, catalase; GPx, glutathione peroxidase; Nrf2, nuclear factor erythroid 2-related factor 2; Keap1, Kelch-like ECH-associated protein 1; TLR4, Toll-like receptor 4; NF-κB, nuclear factor kappa B; MAPK, mitogen-activated protein kinase; PI3K, phosphoinositide 3-kinase; AKT, protein kinase B; mTOR, mechanistic target of rapamycin; AMPK, AMP-activated protein kinase; SIRT1, sirtuin 1; GLUT4, glucose transporter type 4.

The antioxidant effects attributed to *Nymphaea* and *Nelumbo* preparations extend beyond direct radical scavenging and increasingly involve modulation of endogenous signaling networks that regulate antioxidant defense, inflammation, metabolic adaptation, autophagy, and glucose handling. Current mechanistic evidence is concentrated around nuclear factor erythroid 2-related factor 2 (Nrf2)/Kelch-like ECH-associated protein 1 (Keap1)/antioxidant response element (ARE) signaling [9,66,67], Toll-like receptor 4 (TLR4)/nuclear factor kappa B (NF-κB)/mitogen-activated protein kinase (MAPK) signaling [68,69], AMP-activated protein kinase (AMPK)/sirtuin 1 (SIRT1) signaling [70], and phosphoinositide 3-kinase (PI3K)/protein kinase B (AKT)/mechanistic target of rapamycin (mTOR)-dependent autophagy [71]. Alkaloid-mediated effects on glucose handling have additionally been linked to glucose transporter type 4 (GLUT4)-associated mechanisms [72,73]. Importantly, most of this evidence derives from cellular or animal models; pathway modulation should therefore be regarded as preclinical mechanistic evidence rather than proof of therapeutic activity [41,52,74]. The principal reported relationships are summarized in Figure 5.

### 6.1. Nrf2/Keap1/ARE-Mediated Antioxidant Defense

Among the redox-responsive mechanisms investigated in lotus preparations, Nrf2/Keap1/ARE signaling is the most consistently documented. In *N. nucifera*, cellular studies have demonstrated increased nuclear Nrf2 accumulation accompanied by induction of downstream antioxidant defenses. In particular, leaf-derived preparations increased Nrf2-related signaling and enhanced expression of heme oxygenase-1 (HO-1), NAD(P)H quinone dehydrogenase 1 (NQO1), superoxide dismutase 1 (SOD-1), catalase (CAT), and other antioxidant defenses in oxidatively challenged cells [66,75]. Earlier work with a phenolic-rich *N. nucifera* leaf fraction additionally reported marked nuclear Nrf2 accumulation together with increased antioxidant enzyme expression and reduced intracellular ROS and lipid peroxidation, providing an important link between pathway activation and functional cellular protection [66].

The available evidence suggests that structurally different lotus constituents may converge on Nrf2 through different upstream routes. Neferine has been shown to enhance Nrf2 expression and nuclear translocation through modulation of the Keap1–Nrf2 interaction, including competitive binding to Keap1 at Cys-288 in a recent preclinical model [76]. By contrast, proanthocyanidins isolated from *N. nucifera* seed skin have been associated with activation of the p38–Nrf2 axis, induction of downstream antioxidant proteins including HO-1 and NQO1, and concomitant inhibition of NF-κB signaling [67]. These findings support convergent Nrf2 activation through distinct upstream mechanisms rather than a single universal mode of action for lotus phytochemicals. These convergent Nrf2-related mechanisms are illustrated in Figure 5.

Evidence from *Nymphaea* is substantially less developed. *N. nouchali* extracts have been reported to modulate the MAPK/Nrf2/HO-1 axis and decrease intracellular ROS in experimental models [9,77]. This supports participation of Nrf2-related signaling in both genera, but the current evidence is clearly asymmetric. It would therefore be premature to interpret the more extensive *Nelumbo* literature as evidence that *Nelumbo* is intrinsically more effective at activating Nrf2. At present, the stronger mechanistic profile of *N. nucifera* reflects greater experimental depth as much as demonstrated biological differences between the genera.

### 6.2. Inflammatory and Metabolic Redox Signaling

Redox homeostasis is closely coupled to inflammatory and metabolic signaling, and this interaction is particularly apparent in studies of *N. nucifera* alkaloids and polyphenols. Nuciferine has been reported to suppress the TLR4/NF-κB/MAPK axis in preclinical inflammatory models, with accompanying reductions in pro-inflammatory cytokines and restoration of antioxidant defenses [68]. Complementary cellular evidence indicates that nuciferine can also reduce the expression of pro-inflammatory mediators under oxidative stress [78]. Proanthocyanidins from *N. nucifera* seed skin likewise modulate inflammatory and redox signaling, but through a p38/Nrf2/NF-κB-associated mechanism distinct from that described for nuciferine [67]. These findings indicate substantial convergence between inflammatory and redox pathways, but they should not be interpreted as evidence that polyphenols and alkaloids act synergistically within whole lotus extracts. The existing data do not yet justify such a strong conclusion. Different constituent classes clearly converge on overlapping inflammatory signaling nodes, but pathway convergence is not synonymous with pharmacological synergy. Demonstrating synergy would require direct comparison of isolated compounds, defined mixtures, and chemically standardized whole extracts under equivalent experimental conditions.

A second component of this network involves AMPK/SIRT1 signaling, which links metabolic state, mitochondrial function, and redox adaptation. In experimental models of fatty liver and metabolic dysfunction, *N. nucifera* leaf preparations increased hepatic AMPK phosphorylation and SIRT1 expression while reducing lipid accumulation and improving antioxidant defenses [70]. Neferine has also been directly linked to AMPK-associated metabolic signaling, including increased AMPK phosphorylation, GLUT4 expression, and glucose uptake in L6 cells [72,74]. These findings support participation of AMPK-centered signaling in the metabolic actions of *Nelumbo* constituents, although the available evidence remains preclinical.

Nevertheless, AMPK and SIRT1 are pleiotropic stress-responsive regulators rather than lotus-specific molecular targets. Increased AMPK phosphorylation or SIRT1 expression alongside metabolic improvement demonstrates pathway participation but does not establish these proteins as the primary targets responsible for the complete biological response. Taken together, the available evidence is therefore more consistent with modulation of an interconnected inflammatory–metabolic redox network than with selective action on isolated signaling pathways. The interplay between inflammatory, metabolic, and redox signaling is depicted in Figure 5.

### 6.3. Metabolic Homeostasis, Autophagy, and Integrated Redox Responses

PI3K/AKT/mTOR-dependent regulation provides an additional link between metabolic dysfunction, autophagy, and oxidative stress. In an experimental model of diabetic kidney disease, nuciferine treatment restored autophagy-related signaling, including increased Beclin-1 expression and reduced p62 and p-PI3K/p-AKT/p-mTOR levels, accompanied by attenuation of renal injury and fibrosis [71]. These findings support a role for autophagic regulation in the broader protective phenotype attributed to nuciferine. However, they provide stronger evidence for modulation of the PI3K/AKT/mTOR pathway than for a specific direct interaction with mTOR. Claims of direct binding or competitive inhibition should therefore remain provisional unless supported by dedicated biochemical or structural experiments.

Metabolic effects also extend to glucose handling. Neferine increased GLUT4 expression and plasma-membrane translocation in L6 cells through signaling involving protein kinase C (PKC), phospholipase C (PLC), inositol 1,4,5-trisphosphate (IP_3_), and Ca^2+^ mobilization [72]. In parallel, *N. nucifera* seed extracts inhibited both α-amylase and α-glucosidase in biochemical assays [54], while a phenolic-rich lotus seed-skin extract additionally inhibited intestinal α-glucosidase activity and improved glycemic indices in diabetic mice [79]. These mechanisms may contribute to antidiabetic effects reported in experimental models, but they operate at fundamentally different biological levels: digestive-enzyme inhibition modifies carbohydrate availability, GLUT4 regulation affects cellular glucose uptake, and changes in glycemia represent an integrated whole-organism outcome. They should therefore be regarded as complementary mechanisms rather than combined into a single undifferentiated “antidiabetic pathway”. These interconnected mechanisms of redox regulation, autophagy, and glucose handling are integrated in Figure 5.

The physiological relevance of pathway-level findings is supported to some extent by animal studies. In streptozotocin-induced diabetic rats, ethanol extract of *N. nucifera* seeds administered at 200 and 400 mg/kg improved antioxidant status, with the higher dose significantly increasing glutathione peroxidase (GPx), reduced glutathione (GSH), superoxide dismutase (SOD), and catalase (CAT) relative to diabetic controls in serum and selected tissues, including liver and kidney [80]. These findings demonstrate that the redox effects of lotus preparations are not restricted to chemical assays or isolated cellular responses.

The mechanisms considered above are therefore best interpreted as parts of an interconnected preclinical network. Nrf2-dependent antioxidant responses, suppression of NF-κB-related inflammatory signaling, AMPK/SIRT1-mediated metabolic adaptation, mTOR-related autophagy, and regulation of glucose handling may converge on restoration of cellular redox homeostasis. Yet pathway multiplicity should not be mistaken for mechanistic certainty. Much of the current evidence relies on parallel changes in signaling proteins and downstream biomarkers, whereas pathway-specific inhibition, genetic manipulation, pharmacological rescue, and direct target-engagement experiments remain comparatively limited. Moreover, redox responses may be context dependent: a constituent capable of supporting cytoprotection in non-malignant cells may promote ROS-associated pro-apoptotic responses in cancer models [81,82], precluding a universal classification of lotus constituents as simply “antioxidant”.

Accordingly, the mechanistic framework for *Nymphaea* and *Nelumbo* is biologically coherent but only partially validated. The strongest evidence supports modulation of interconnected redox-responsive pathways; it does not yet establish a complete causal map linking individual phytochemicals, molecular targets, systemic exposure, and therapeutic outcomes.

## 7. From Preclinical Evidence to Clinical Translation

The mechanistic evidence summarized in Section 6 is substantial, but it remains predominantly preclinical. Accordingly, the following section moves beyond pathway-level interpretation to consider the translational constraints imposed by bioavailability, metabolism, pharmacokinetics, formulation, and standardization. Cellular and animal studies support modulation of several redox-, inflammatory-, and metabolism-related pathways; however, these findings do not establish that equivalent target engagement can be achieved in humans at realistic oral exposures. This distinction is central to the translational interpretation of lotus-derived preparations and is summarized in Table 3.

The evidence map reveals a marked imbalance between mechanistic plausibility and translational validation. Most reported effects arise from in vitro or animal studies, whereas controlled human evidence using chemically standardized preparations remains very limited [41]. Differences in species, plant organ, extraction procedure, dose, and phytochemical composition further weaken direct comparison across studies.

Oral bioavailability is a major determinant of whether biological effects observed in vitro can be reproduced in vivo. Nuciferine undergoes extensive phase I and phase II metabolism, including demethylation and oxidation as well as glucuronidation and sulfation [84,85], whereas neferine metabolism involves demethylation, dealkylation, dehydrogenation, and glucuronidation [86]. These metabolic transformations, together with the relatively limited in vivo bioavailability reported for lotus alkaloids, may constrain systemic exposure to the parent compounds [41].

Formulation strategies have begun to address the limited oral exposure of selected lotus alkaloids. In particular, nuciferine-loaded poly(lactic-co-glycolic acid) (PLGA) nanoparticles improved aqueous solubility, sustained release, and oral bioavailability relative to free nuciferine in experimental models [87]. Polyphenols introduce an additional layer of complexity because intestinal and microbial metabolism can convert poorly absorbed parent compounds into lower-molecular-weight metabolites with distinct absorption and biological properties [88]. Consequently, the compounds dominating the administered extract may not be those primarily responsible for systemic effects after oral consumption. Improved delivery therefore has translational value only when enhanced exposure can be linked to target engagement and a reproducible biological response.

This exposure problem cannot be separated from the chemical heterogeneity of the preparations themselves. Differences in plant organ, geographic origin, sample condition, extraction procedure, and processing can generate markedly different phytochemical exposures even within the same botanical species [51,56]. Clinically oriented preparations should therefore combine verified botanical identity with defined plant organ and extraction conditions, quantitative characterization of relevant alkaloid and flavonoid markers, demonstrated batch reproducibility, stability assessment, and appropriate contaminant control.

Regulatory standardization of botanical preparations remains challenging because their inherent chemical complexity and variability require control of botanical identity, starting materials, manufacturing conditions, and batch-to-batch consistency. Current regulatory guidance therefore emphasizes comprehensive specifications and quality-control strategies rather than reliance on a single marker compound [89,90]. Preparations standardized only to nuciferine, neferine, or total phenolics may still differ substantially in minor alkaloids, flavonoid conjugates, and other constituents capable of modifying efficacy, pharmacokinetics, or safety.

The translational bottleneck therefore lies at the intersection of exposure and reproducibility: the biologically relevant chemical species must reach effective concentrations, while the administered preparation must be sufficiently standardized for those exposures to be comparable across studies and, ultimately, across patients. Human pharmacokinetic studies integrating parent compounds, metabolites, and exposure–response relationships, together with comprehensive chemical fingerprinting of the administered material, are therefore essential for meaningful clinical translation.

## 8. Safety Considerations

Safety assessment of *Nymphaea* and *Nelumbo* requires a distinction between whole botanical preparations, enriched fractions, and isolated alkaloids. The available evidence is uneven across species and exposure durations: acute rodent studies are relatively common, whereas repeated-dose, reproductive, interaction, and human safety data remain much more limited. This imbalance is important because favorable acute tolerance cannot be extrapolated directly to long-term consumption of concentrated extracts or alkaloid-rich supplements.

### 8.1. Acute and Repeated-Dose Toxicity

Acute oral toxicity studies generally indicate relatively low acute toxicity for several botanical preparations. In rats, *N. nucifera* stamen extract produced no treatment-related signs of toxicity or mortality following a single oral dose of 5000 mg/kg [91], while a hydroalcoholic rhizome extract produced no acute toxic effects at 2000 mg/kg [92]. Similarly, aqueous *N. lotus* leaf and flower preparations showed no mortality or overt acute toxicity at doses up to 5000 mg/kg [93,94]. These findings support a comparatively wide acute safety margin for the tested extracts, but they should not be generalized across plant organs, extraction procedures, or purified constituents.

The distinction becomes particularly important for alkaloid-enriched materials. Historical pharmacological data for isolated nuciferine indicate oral median lethal dose (LD_50_) values of approximately 240 mg/kg in mice and 280 mg/kg in rats [95], markedly lower than the doses tolerated for several crude botanical preparations. These historical values remain relevant as primary pharmacological data, although contemporary assessments emphasize that the safety of lotus alkaloids remains incompletely characterized and should be interpreted in relation to compound identity, dose, exposure, and pharmacokinetic behavior [41]. This difference should not be interpreted as evidence of an intrinsic protective effect of the whole extract, because dose, route of administration, alkaloid concentration, and matrix composition differ substantially among studies. Whole-extract LD_50_ values therefore provide limited information about the safety of concentrated alkaloid preparations.

Repeated-dose studies are more relevant to nutraceutical use and reveal a less uniform picture. *N. nucifera* stamen extract administered orally for 90 days at 50–200 mg/kg/day produced no gross or histopathological abnormalities, although some statistically significant hematological and biochemical changes were observed at the highest dose; the reported no-observed-adverse-effect level (NOAEL) was 200 mg/kg/day [91]. A 28-day study of *N. nucifera* rhizome extract at 250–1000 mg/kg likewise reported no major treatment-related toxicity [92]. In *N. lotus*, however, repeated administration of leaf extracts produced selected biochemical changes, including alterations in renal-related parameters, despite the absence of clear treatment-related histopathological lesions [93,96]. These findings illustrate why favorable acute tolerance should not be extrapolated directly to repeated or chronic exposure.

These findings should nevertheless be interpreted conservatively. Differences among preparations, doses, plant organs, and exposure durations prevent a simple ranking of species as “safe” or “unsafe”. More importantly, the evidence base is considerably stronger for acute and short-term tolerance than for chronic safety, which remains poorly characterized for several preparations of current commercial interest. Reproductive and developmental safety remains substantially less characterized. Available experimental studies have primarily examined reproductive function in disease or toxicant-associated models rather than dedicated developmental-toxicity, pregnancy, or multigenerational designs. Such findings cannot establish the safety of concentrated lotus extracts or isolated alkaloids during pregnancy or lactation. Reproductive and developmental safety should therefore be regarded as an unresolved evidence gap rather than a demonstrated area of safety.

### 8.2. Herb–Drug Interaction Potential

Potential herb–drug interactions represent a distinct safety concern, particularly for alkaloid-rich *N. nucifera* preparations. In human liver microsomes, lotus leaf alcoholic extract and its alkaloid fraction inhibited cytochrome P450 2D6 (CYP2D6) activity, while isolated aporphine alkaloids including nuciferine and N-nornuciferine showed competitive CYP2D6 inhibition [97]. Nuciferine has also been reported to inhibit cytochrome P450 1A2 (CYP1A2) and to alter the pharmacokinetics of the CYP1A2 probe substrate phenacetin in rats [98]. These findings establish interaction potential at the preclinical level, although their clinical relevance remains dependent on oral exposure, extract composition, and alkaloid concentration.

Pharmacodynamic interaction potential is likewise biologically plausible. Nuciferine displays activity across several serotonergic and dopaminergic receptors [99], whereas neferine has been linked to 5-hydroxytryptamine 1A (5-HT_1_A) receptor-mediated modulation of neurotransmitter release [100]. In addition, aporphine alkaloids isolated from *N. nucifera* leaves, including nuciferine and nornuciferine derivatives, inhibited hERG potassium channels in vitro, and their abundance varied markedly among commercial lotus-containing supplements [101]. These observations justify caution with concentrated alkaloid-rich preparations, particularly during concomitant pharmacotherapy, but they do not establish specific clinical interaction syndromes.

**Figure 6:**
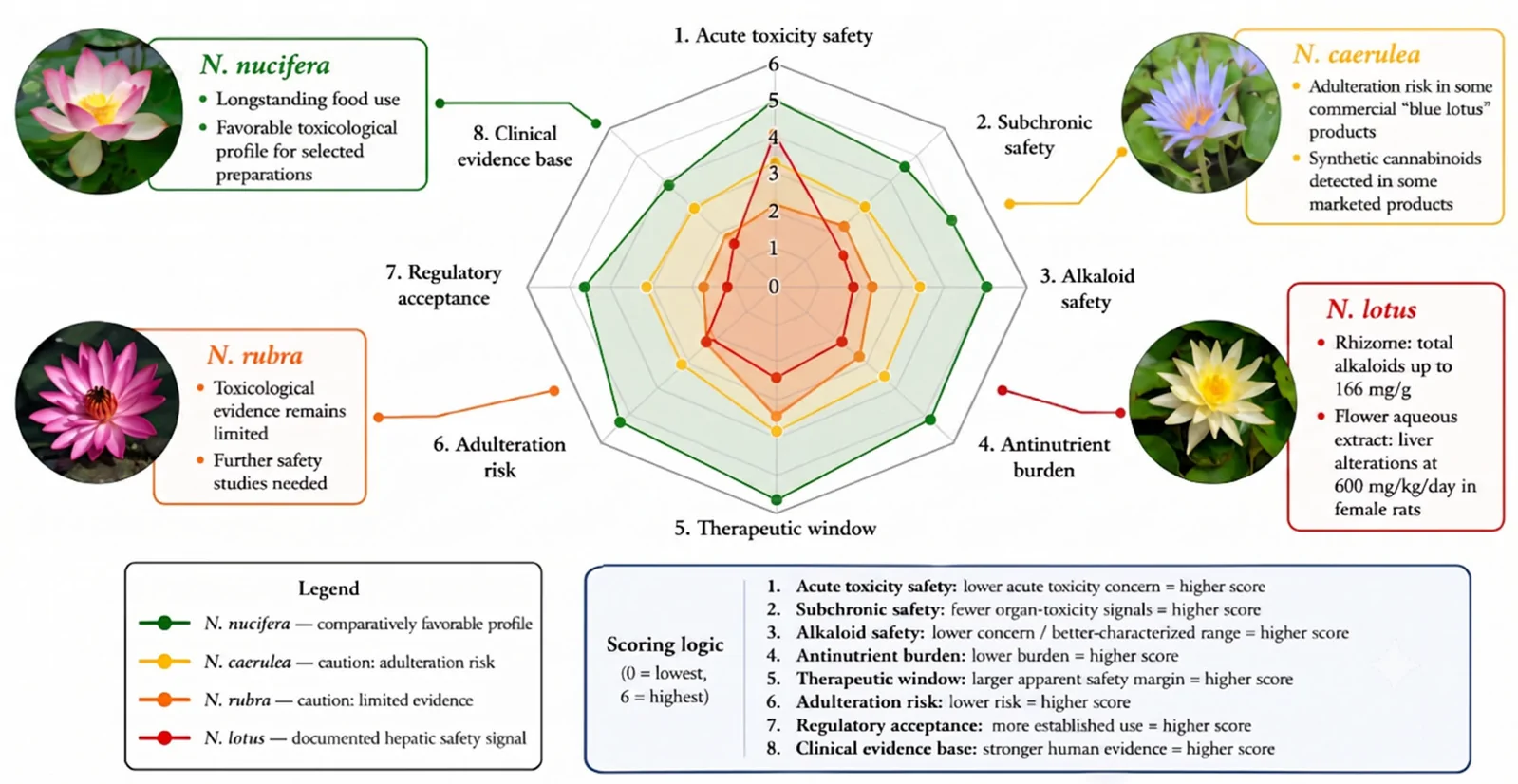
Qualitative evidence-informed safety profile of selected *Nymphaea* and *Nelumbo* species. The radar plot summarizes eight safety-related domains, integrating reported toxicological findings with the relative maturity of the available evidence. Scores are qualitative and comparative; higher values generally indicate a more favorable or better-supported profile within the respective domain and should not be interpreted as validated toxicological, clinical, or regulatory risk estimates.

Overall, the current evidence supports interaction potential more strongly than clinically demonstrated interaction risk. Controlled human interaction studies with chemically standardized lotus preparations are lacking, and the broader safety evidence remains highly uneven across species, preparations, and exposure scenarios. Acute tolerance is comparatively well documented for several botanical extracts, whereas repeated-dose, reproductive, interaction, and clinical safety remain substantially less developed. Importantly, the absence of a reported toxicity signal should not be interpreted as evidence of safety when the underlying evidence base is limited. Safety assessment should therefore remain species-, organ-, preparation-, and exposure-specific and should consider botanical identity, extract composition, alkaloid content, dose, and achievable systemic exposure, with particular caution applied to concentrated alkaloid-rich products and commercially adulterated materials. This heterogeneity is illustrated in Figure 6, which integrates reported safety signals with the relative maturity of the available evidence across selected toxicological, interaction, regulatory, and clinical domains.

The radar profile should be interpreted primarily as a visualization of both safety-related findings and evidence maturity rather than as a ranking of intrinsic species safety. A broader profile may reflect a combination of more favorable findings and a substantially more developed experimental evidence base, whereas a smaller profile may result from insufficient characterization rather than greater toxicological risk. This distinction is particularly relevant for *Nymphaea* species, for which repeated-dose, reproductive, interaction, and clinical safety data remain comparatively sparse. Conversely, the broader profile of *N. nucifera* reflects, at least in part, the much greater depth of its toxicological and pharmacological characterization. For *N. caerulea*, interpretation is further complicated by the documented adulteration and uncertain composition of some commercial “blue lotus” products [2,29], meaning that adverse effects reported for marketed products cannot always be attributed confidently to authenticated plant material. Overall, the available evidence supports a precautionary, species-, organ-, preparation-, and exposure-specific approach rather than a single safety designation for materials marketed generically as “lotus”.

## 9. Research Priorities and Future Directions

The next phase of research on *Nymphaea* and *Nelumbo* should move beyond the accumulation of isolated antioxidant results and pathway associations. The central challenge is now to connect botanical identity, chemical composition, biological exposure, mechanistic response, and safety within the same experimental framework.

First, authentication and chemical standardization must become integral to study design rather than post hoc quality-control steps. Species identity, plant organ, geographic origin, extraction procedure, and quantitative phytochemical composition should be reported systematically, supported where appropriate by voucher specimens, molecular identification, and chemical fingerprinting [28,102]. Without this, apparent differences in efficacy or safety may continue to reflect material heterogeneity rather than true biological variation.

Second, translational studies should prioritize pharmacokinetic relevance. Human efficacy cannot be inferred from in vitro potency unless the relevant parent compounds or active metabolites reach biologically meaningful concentrations after oral administration. This is particularly important for nuciferine, neferine, and extensively metabolized polyphenols, as well as for microbiota-derived metabolites whose contribution to systemic activity remains insufficiently defined.

Safety assessment must progress in parallel with efficacy. Acute toxicity data are insufficient to characterize repeated exposure, concentrated extracts, alkaloid-enriched preparations, vulnerable populations, or clinically relevant interaction risks. Future toxicological and pharmacological studies should therefore use the same authenticated and chemically characterized preparations.

Finally, omics-based approaches should be used selectively to connect biosynthetic regulation with quantitative chemistry and biological response rather than to generate additional descriptive datasets. The field does not primarily need more evidence that lotus preparations are biologically active; it needs better-integrated evidence showing which authenticated preparation, at what exposure, produces which effect, through which mechanism, and with what safety margin.

## 10. Conclusions

The available evidence does not support treating Nymphaea and Nelumbo as interchangeable “lotus” materials. Despite their shared ethnobotanical designation and broadly overlapping antioxidant potential, the two genera differ fundamentally in taxonomy, biosynthetic organization, phytochemical composition, and the maturity of their pharmacological and safety evidence. Botanical authentication is therefore not merely a taxonomic requirement, but a prerequisite for meaningful interpretation of efficacy and risk.

Among the investigated species, *Nelumbo nucifera* has the most extensively characterized phytochemical and mechanistic profile, particularly with respect to phenolics and benzylisoquinoline alkaloids. By contrast, evidence across *Nymphaea* remains more heterogeneous and species dependent. Importantly, neither high phenolic content nor the detection of pharmacologically relevant alkaloids is sufficient to predict biological efficacy. Chemical composition must be considered together with plant organ, extraction procedure, bioavailability, metabolism, and achievable systemic exposure.

The major weakness of the current evidence base is therefore translational rather than mechanistic. Multiple redox-, inflammatory-, and metabolism-related pathways have been implicated in preclinical models, yet their causal relevance and reproducibility in humans remain insufficiently established. Safety is similarly unevenly characterized, and favorable acute tolerance should not be interpreted as evidence of long-term safety, particularly for concentrated or poorly authenticated commercial preparations.

The field now needs fewer isolated demonstrations of “antioxidant activity” and more integrated evidence linking authenticated botanical material, defined chemical composition, systemic exposure, target engagement, clinically meaningful outcomes, and safety. Until this chain is established, the therapeutic potential of *Nymphaea* and *Nelumbo* should be regarded as scientifically plausible and pharmacologically promising, but not clinically validated.

## Figures and Tables

**Table 1:** Comparative organ-specific phytochemical profiles of selected *Nymphaea* taxa and *Nelumbo nucifera*.

Taxon	Organ/Material	TPC (Reported Basis)	TFC (Reported Basis)	Major Phytochemicals/Profile	Quantitative/Diagnostic Information	Representative References
***Nymphaea nouchali var. caerulea*** **(syn. *N. caerulea*)**	Flower (80% methanolic extract)	561.5 ± 15.1 mg GAE/g extract	116.11 ± 7.69 mg QE/g extract	Quercetin-, kaempferol-, myricetin-, patuletin- and isorhamnetin-derived glycosides; gallic-acid derivatives; ellagitannins	185 metabolites annotated; 46 flavonol glycosides	[7,11]
***Nymphaea*** ***caerulea***	Flower concrete/absolute (authenticated industrial extracts)	n.r.	n.r.	Nuciferine; apomorphine	Nuciferine 10–72 ppb; apomorphine not detected	[2]
***Nymphaea*** ***lotus***	Rhizome (methanolic extract)	n.r.	n.r.	Total alkaloids; flavonoids; tannins; saponins (class-level assays)	166.0 ± 0.57 mg/g total alkaloids; 80.0 ± 0.29 mg/g flavonoids; 90.0 ± 0.50 mg/g tannins; 22.0 ± 0.12 mg/g saponins	[47]
***Nymphaea*** ***lotus***	Stamen and perianth (13 natural populations)	n.r.	n.r.	Kaempferol 3-O-galactoside; quercetin 3′-O-xyloside; quercetin 3-O-rhamnoside; isorhamnetin 7-O-galactoside; myricetin 3′-O-xyloside	Individual flavonoids: 3.1–137.3 mg/100 g DW across floral parts and populations	[37]
***Nymphaea*** ***rubra***	Leaf (optimized ultrasound-assisted extract)	45.12 ± 1.12 mg GAE/g	44.05 ± 1.65 mg CE/g	Broad phenolic profile including flavonoids, tannins and other secondary metabolites	150 secondary metabolites annotated by high-resolution MS	[46]
***Nymphaea*** ***alba***	Leaf extract	n.r.	n.r.	Hydrolysable tannins; gallic- and ellagic-acid derivatives; flavonoids including quercetin 3-O-acetyl hexoside	53 constituents characterized: 42 hydrolysable tannins, 3 simple phenolic acids and 8 flavonoids	[48]
***Nelumbo*** ***nucifera***	Old leaf (aqueous extract; three harvest periods)	5.11–38.85 mg GAE/g DW	n.r.	Quercetin 3-O-glucuronide predominant in leaf extracts; diverse flavonoid and alkaloid profile	Separate HPLC-QTOF study identified 30 compounds (12 flavonoids, 16 alkaloids, 2 proanthocyanidins)	[25,53]
***Nelumbo*** ***nucifera***	Seed (methanolic ultrasound-assisted extract)	107.92 mg GAE/g extract	104.89 mg QE/g extract	*p*-Coumaric acid; chlorogenic acid	*p*-Coumaric acid 5.60 mg/g; chlorogenic acid 4.27 mg/g	[54]
***Nelumbo*** ***nucifera***	Seed embryo/plumule	10.77 ± 0.66 mg GAE/g FW	1.61 ± 0.03 mg QE/g FW	C-glycosylated flavonoids and phenolic acids; bisbenzylisoquinoline alkaloids (neferine, liensinine, isoliensinine)	59 phenolic compounds identified in embryo; plumule alkaloids: neferine 4.68–6.56 mg/g, liensinine 0.72–2.13 mg/g, isoliensinine 1.47–2.95 mg/g	[55,56]
***Nelumbo*** ***nucifera***	Rhizome (pulp/peel/knot)	0.34 ± 0.01/3.44 ± 0.07/3.49 ± 0.12 mg GAE/g FW	0.01 ± 0.01/0.31 ± 0.02/0.24 ± 0.02 mg QE/g FW	Phenolic acids and flavonoids; strong tissue-part dependence	Values correspond respectively to pulp/peel/knot	[56]
***Nelumbo*** ***nucifera***	Flower extract fractions (water; 30%, 70% and 100% ethanol fractions)	14.12–137.45 mg GAE/g dry extract	6.81–440.15 mg RE/g dry extract	Myricetin-, quercetin- and kaempferol-derived glycosides	Marked fraction-dependent enrichment of phenolics and flavonoids	[50]

**Notes.** Values are reported as provided in the original studies and should not be interpreted as directly comparable across taxa or organs, because extraction procedures, sample basis (fresh weight, dry weight or dry extract), calibration standards and analytical platforms differed. Where two analytical datasets are summarized in one row, this is stated explicitly. n.r., not reported in the cited study; GAE, gallic acid equivalents; QE, quercetin equivalents; CE, catechin equivalents; RE, rutin equivalents; FW, fresh weight; DW, dry weight.

**Table 2:** Comparative antioxidant activities reported for selected *Nymphaea* and *Nelumbo* extracts across complementary assay systems.

Species	Organ/Material	Extraction Method	DPPH	ABTS	FRAP	TPC (Reported Basis)	TFC (Reported Basis)	Reference
***N. nouchali var. caerulea*** **(syn. *N. caerulea*)**	Flower	80% methanolic extract	IC_50_ 45.15 μg/mL	IC_50_ 5.02 μg/mL	n.r.	561.5 ± 15.1 mg GAE/g extract	116.11 ± 7.69 mg QE/g extract	[7]
***N. rubra***	Leaf	Optimized UAE (53% ethanol)	19.21 ± 1.71 μM ASCE/g	24.59 ± 1.35 μM ASCE/g	61.32 ± 2.56 μM ASCE/g	45.12 ± 1.12 mg GAE/g	44.05 ± 1.65 mg CE/g	[46]
***N. lotus***	Stamen	UAE + DAX-8 macroporous-resin enrichment	4749.67 ± 166.05 μmol TEAC/g	4710.67 ± 155.64 μmol TEAC/g	3601.74 ± 126.26 μmol TEAC/g	n.r.	475.42 ± 16.61 mg/g DW	[62]
***N. nucifera***	Stamen/perianth (18 populations)	UAE, 90% aqueous ethanol	S: 96.4–204.5; P: 85.3–139.3 μmol TE/g DW	S: 27.4–60.5; P: 24.0–40.4 μmol TE/g DW	S: 210.0–319.6; P: 49.3–162.7 μmol TE/g DW	S: 492.1–669.4; P: 95.2–233.9 mg GAE/100 g DW	S: 570.6–1684.3; P: 128.1–315.7 mg QE/100 g DW	[37]
***N. nucifera***	Flower, 30% ethanol fraction	Sequential fractionation of flower water extract	IC_50_ 8.20 ± 0.50 μg/mL	IC_50_ 11.70 ± 0.15 μg/mL	IC_50_ 140.11 ± 1.08 μg/mL	137.45 ± 2.65 mg GAE/g DE	102.85 ± 29.19 mg RE/g DE	[50]
***N. nucifera***	Seed embryo	80% ethanol extract	9.66 ± 0.10 mg AAE/g FW	14.35 ± 0.20 mg AAE/g FW	1.72 ± 0.02 mg AAE/g FW	10.77 ± 0.66 mg GAE/g FW	1.61 ± 0.03 mg QE/g FW	[56]
***N. nucifera***	Leaf phenolic fraction (EAF)	Methanolic extract → ethyl acetate fraction	IC_50_ 4.46 ± 0.01 μg/mL	IC_50_ 5.35 ± 0.02 μg/mL	n.r. (reducing power reported separately)	346.26 ± 15.22 mg GAE/g EAF	115.86 ± 1.12 mg QE/g EAF	[63]

**Notes.** Values are reported as provided in the original studies and are not directly comparable across taxa or extracts because assay format, extraction procedure, calibration standard, sample basis, and reporting units differed among studies. n.r., not reported; S, stamen; P, perianth; UAE, ultrasound-assisted extraction; MPR, macroporous resin; GAE, gallic acid equivalents; QE, quercetin equivalents; CE, catechin equivalents; RE, rutin equivalents; ASCE, ascorbic acid standard equivalents; AAE, ascorbic acid equivalents; TE/TEAC, Trolox equivalents; DE, dry extract; DW, dry weight; FW, fresh weight; EAF, ethyl acetate fraction; IC_50_, concentration required for 50% inhibition; DPPH, 2,2-diphenyl-1-picrylhydrazyl; ABTS, 2,2′-azino-bis(3-ethylbenzothiazoline-6-sulfonic acid); FRAP, ferric reducing antioxidant power; TPC, total phenolic content; TFC, total flavonoid content.

**Table 3:** Mapping of Reported Molecular Mechanisms to Preclinical Outcomes.

Pathway	Preclinical Outcome	Mechanistic Evidence	In Vivo/Ex Vivo Evidence
**Nrf2/Keap1/ARE**	Antioxidant and cytoprotective effects	*N. nucifera* leaf preparations increased nuclear Nrf2 and downstream antioxidant defenses in oxidatively challenged hepatocytes [66,75]; *N. nouchali* extracts modulated MAPK/Nrf2/HO-1 signaling and reduced ROS [9,77].	*N. nucifera* seed extract improved GPx, GSH, SOD, and CAT in streptozotocin-diabetic rats [80].
**TLR4/NF-κB/MAPK**	Anti-inflammatory effects	Nuciferine suppressed TLR4/NF-κB/MAPK signaling and pro-inflammatory cytokines [68]; lotus seed-skin proanthocyanidins modulated p38/Nrf2/NF-κB signaling [67].	Nuciferine reduced arthritis severity and inflammatory markers in adjuvant-induced arthritis in rats [68].
**AMPK/GLUT4** **signaling**	Antidiabetic effects: glucose uptake and glycemic regulation	Neferine increased AMPK phosphorylation, GLUT4 expression, and plasma-membrane translocation in L6 cells [72].	Direct in vivo confirmation of GLUT4 pathway engagement remains limited; *N. nucifera* seed extract improved glycemic endpoints in diabetic rats [80].
**Enzyme inhibition: α-glucosidase and α-amylase**	Antidiabetic effects: postprandial carbohydrate handling	*N. nucifera* seed and seed-skin extracts inhibited α-amylase and/or α-glucosidase in biochemical assays [54,79].	Phenolic-rich lotus seed-skin extract improved glycemic indices in diabetic mice [79].
**AMPK/SIRT1** **and Nrf2/HO-1**	Hepatoprotective effects: non-alcoholic fatty liver disease	Lotus leaf treatment modulated AMPK/SIRT1 and Nrf2/HO-1 signaling in the reported experimental model [70].	In high-fat-diet rats, lotus leaf reduced hepatic triglyceride accumulation and improved histopathology [70].
**Nrf2/HO-1 and** **oxidative-stress modulation**	Hepatoprotective effects: toxin-associated injury	*N. nucifera* leaf phenolic fraction activated HO-1/Nrf2 and antioxidant enzymes in oxidatively stressed hepatocytes [66].	*N. nucifera* petal-tea extract reduced mancozeb-associated hepatic oxidative stress and injury in rats [83].
**PI3K/AKT/mTOR-dependent** **autophagy**	Renoprotective effects: diabetic kidney disease and fibrosis	Nuciferine restored autophagy-related signaling, with increased Beclin-1 and reduced p62 and p-PI3K/p-AKT/p-mTOR in experimental diabetic kidney disease [71].	Nuciferine reduced renal fibrosis and related injury endpoints in the same preclinical model [71].

*Note.* All evidence summarized in this table derives from biochemical assays, cultured cells, ex vivo preparations, or animal models. No controlled human trial of a chemically standardized *Nymphaea* or *Nelumbo* preparation was identified in the literature search for the stated outcomes. Abbreviations: Nrf2, nuclear factor erythroid 2-related factor 2; Keap1, Kelch-like ECH-associated protein 1; ARE, antioxidant response element; TLR4, Toll-like receptor 4; NF-κB, nuclear factor kappa B; MAPK, mitogen-activated protein kinase; AMPK, AMP-activated protein kinase; GLUT4, glucose transporter type 4; SIRT1, sirtuin 1; HO-1, heme oxygenase-1; PI3K, phosphoinositide 3-kinase; AKT, protein kinase B; mTOR, mechanistic target of rapamycin; GPx, glutathione peroxidase; GSH, reduced glutathione; SOD, superoxide dismutase; CAT, catalase.

## Data Availability

No new data were created or analyzed in this study. Data sharing is not applicable to this article.
